# 预后营养指数在晚期NSCLC伴恶性胸腔积液患者预后和胸膜自发固定中的预测价值

**DOI:** 10.3779/j.issn.1009-3419.2024.106.33

**Published:** 2024-12-20

**Authors:** Sihan TAN, Weimin LI, Panwen TIAN

**Affiliations:** 610041 成都，四川大学华西医院呼吸与危重症医学科/肺癌中心，呼吸和共病全国重点实验室，精准医学四川省重点实验室; Department of Pulmonary and Critical Care Medicine/Lung Cancer Institute, State Key Laboratory of Respiratory Health and Multimorbidity, Precision Medicine Key Laboratory of Sichuan Province, West China Hospital, Sichuan University, Chengdu 610041, China

**Keywords:** 肺肿瘤, 恶性胸腔积液, 预后营养指数, 预后, 胸膜自发固定, Lung neoplasms, Malignant pleural effusion, Prognostic nutritional index, Prognosis, Spontaneous pleurodesis

## Abstract

**背景与目的:**

基于营养状况和炎症的预后营养指数（prognostic nutritional index, PNI）已被开发并证明与恶性肿瘤不良预后密切相关。然而，PNI在恶性胸腔积液（malignant pleural effusion, MPE）患者中的预测作用仍不确定。本研究旨在探究PNI在MPE患者预后和胸膜自发固定中的预测价值。

**方法:**

回顾性分析2015年1月至2022年12月于四川大学华西医院诊断为晚期非小细胞肺癌（non-small cell lung cancer, NSCLC）伴MPE患者病例资料，并将患者随机分配到训练集（60%）和验证集（40%）。收集患者的临床数据和外周血炎症指标并计算全身炎症系数，使用Cox比例风险模型、Kaplan-Meier法、Nelson-Aalen累积风险曲线分析PNI对MPE患者预后和胸膜自发固定的影响。

**结果:**

本研究最终纳入261例晚期NSCLC伴MPE患者（训练集157例，验证集104例），其中年龄<65岁占58.2%，男性患者占53.6%，腺癌占95.8%。PNI的二分类截断值为44.1，与PNI较低（PNI<44.1）的患者相比，PNI高（PNI≥44.1）的患者中位生存期明显延长（36.5 vs 24.3个月，*P*=0.02），胸膜自发固定发生率更高（*P*=0.009）。多因素Cox分析发现，较高的PNI是患者良好预后和胸膜自发固定的独立影响因素（*P*<0.05）。根据Cox回归分析结果确定PNI-预后和PNI-胸膜自发固定预测模型，绘制受试者工作特征（receiver operating characteristic, ROC）曲线，训练集的曲线下面积（area under the curve, AUC）分别为0.694（95%CI: 0.620-0.776）和0.673（95%CI: 0.590-0.737）。

**结论:**

PNI是衡量MPE患者预后和胸膜自发固定的可靠生物标志物，关注患者的营养状况和免疫状态可能会改善患者的预后和胸腔积液的控制。

## Abstract (en)

**Background and objective:**

A prognostic nutritional index (PNI) developed by nutritional status and inflammation are closely associated with poor prognosis in malignant tumors. However, the predictive impact of PNI in patients with malignant pleural effusion (MPE) remains inconclusive. The study aimed to determine the predictive value of PNI in prognosis and spontaneous pleurodesis among patients with MPE.

**Methods:**

The patients diagnosed with advanced non-small cell lung cancer (NSCLC) with MPE in West China Hospital between January 2015 and December 2022 were reviewed and allocated randomly to development set(60%) and validation set(40%). After collecting clinical data, peripheral blood inflammation indices and calculating systemic inflammation indices, the effects of PNI on prognosis and spontaneous pleurodesis have been evaluated by Cox proportional hazards models, Kaplan-Meier method and Nelson-Aalen cumulative risk curve.

**Results:**

In total, 261 patients diagnosed NSCLC with MPE were selected (development set: *n*=157; validation set: *n*=104), of whom 58.2% were aged <65 years, 53.6% were male and 95.8% were diagnosed with adenocarcinoma. The dichotomous cut-off value for PNI was 44.1, respectively. Compared with lower PNI (PNI<44.1) cases, patients with higher PNI (PNI≥44.1) showed significantly longer overall survival (36.5 vs 24.3 mon, *P*=0.02) and higher incidence of spontaneous pleurodesis (*P*=0.009). According to the multivariate Cox analysis, higher PNI was associated with good prognosis and successful spontaneous pleurodesis (*P*<0.05). According to the results of Cox regression analysis, the PNI-prognosis and PNI-spontaneous pleurodesis models are determined, the receiver operating characteristic (ROC) curves are drawn, and the area under the curves (AUC) value of development set are 0.694 (95%CI: 0.620-0.776) and 0.673 (95%CI: 0.590-0.737).

**Conclusion:**

PNI is a reliable biomarker of prognosis and spontaneous pleurodesis in patients with MPE. Attention to the patient's nutritional status and inflammation may improve the prognosis and efficacy of pleural effusion.

恶性胸腔积液（malignant pleural effusion, MPE）是由原发性或继发性恶性肿瘤累及胸膜所引起，其中肺癌是最常见的病因，约占40%^[1]^。MPE患者常伴有进行性呼吸困难、咳嗽、疼痛等症状，生活质量降低，总生存期（overall survival, OS）缩短^[1⇓-3]^。近年来，由于全身和局部治疗的新技术和新方案的研发和应用，MPE患者的预后和胸腔积液控制得到了改善，同时患者的临床管理和预后模式可能变得复杂和不同。其中预后较好的患者可能更适宜接受更积极、侵入性的治疗，而对于生存期有限的患者，更适宜接受胸腔穿刺等支持治疗^[4]^。对于MPE胸膜自发固定成功率低的患者，滑石粉化学胸膜固定及新的胸腔介入技术，将有可能提高患者胸膜固定率和生活质量^[5,6]^。因此，临床医师有必要探讨MPE患者不良预后和胸膜自发固定的影响因素，为制定治疗方案和后续临床管理提供理论依据。

既往研究^[7]^表明长期营养不良与肿瘤患者较差的预后相关，有10%-20%肿瘤患者因营养不良而发生非肿瘤相关死亡^[8]^。此外，越来越多的研究^[9,10]^表明全身炎症在癌症发生和发展中发挥重要的作用，外周血免疫细胞与肿瘤的增殖和侵袭有关^[11,12]^。因此，研究者们开发并应用炎症复合指标预测恶性肿瘤患者的预后，包括晚期肺癌炎症指数（advanced lung cancer inflammation index, ALI）^[13]^、预后营养指数（prognostic nutritional index, PNI）^[14]^、基线血红蛋白、白蛋白、淋巴细胞和血小板评分（hemoglobin, albumin, lymphocyte, and platelet, HALP）^[15]^和全身性免疫炎症指数（systemic immune inflammation index, SII）^[16]^等。然而，上述营养和全身炎症指标在MPE中尚缺少相关研究。

因此，本研究拟探讨不同的营养和全身炎症指标如ALI、PNI、HALP和SII在MPE患者预后和胸膜自发固定中的作用，并确定影响患者预后及自发胸膜固定的因素。

## 1 资料与方法

### 1.1 病例来源

回顾性收集2015年1月至2022年12月于四川大学华西医院初诊为晚期非小细胞肺癌（non-small cell lung cancer, NSCLC）伴MPE患者的临床资料。纳入标准：（1）年龄≥18岁；（2）组织病理确诊为NSCLC；（3）病理或临床诊断MPE，满足以下任意一条：①病理确诊恶性肿瘤，PE难以用其他病因解释；②病理确诊恶性肿瘤，存在PE，影像学即计算机断层扫描（computed tomography, CT）或正电子发射断层显像（positron emission tomography, PET）/CT或磁共振成像（magnetic resonance imaging, MRI）或胸部超声提示胸膜结节或肿块或不规则增厚，符合胸膜转移影像学特征；（4）确诊后至少规律治疗2个周期；（5）接受胸腔置管引流治疗。排除标准：（1）临床资料严重缺失，无随访记录；（2）合并其他部位原发肿瘤病史；（3）合并免疫性疾病；（4）无法评估胸膜自发固定的发生情况；（5）治疗中出现MPE；（6）接受胸腔穿刺、胸腔内抗肿瘤治疗、胸腔热灌注化疗、胸腔内介入治疗（电刀、冷冻、激光等）、外科胸膜切除术治疗。本研究回顾了387例晚期NSCLC伴MPE病例资料，排除了55例未接受胸腔置管引流、28例接受胸腔内抗肿瘤治疗和43例临床及随访数据不完整的患者，最终纳入261例初诊晚期NSCLC伴MPE并接受胸腔置管引流的患者，按照6:4的比例分为训练集和验证集。本研究得到四川大学华西医院伦理委员会批准（No.2024-197）。

### 1.2 资料收集

收集患者的一般临床资料，包括年龄、性别、吸烟史、东部合作肿瘤小组（Eastern Cooperative Oncology Group, ECOG）体能状态评分、肺癌家族史、影像学、治疗方案及随访记录等。收集治疗前7天以内血常规、血生化、凝血指标等检验数据。所有患者都接受了基于DNA的下一代测序（next-generation sequencing, NGS）和免疫组化检查，以检测表皮生长因子受体（epidermal growth factor receptor, EGFR）突变或间变性淋巴瘤激酶（anaplastic lymphoma kinase, ALK）/肉瘤致癌因子-受体酪氨酸激酶（ROS proto-oncogene 1, receptor tyrosine kinase, ROS1）融合，样本来源包括组织学标本和MPE样本。

### 1.3 治疗及随访

肺癌临床分期根据第八版肺癌原发灶-淋巴结-转移（tumor-node-metastasis, TNM）分期进行评估，EGFR敏感突变、ALK/ROS1融合的患者接受相应靶向治疗，驱动基因阴性的患者接受化疗联合或不联合免疫治疗。每8-10周使用胸、腹部CT和头部MRI评估肿瘤的疗效。MPE 的评估基于胸部CT或胸部彩超。通过电子病历或电话进行随访，末次随访时间为2024年1月。主要的结局指标为OS，定义为接受一线治疗开始到因任何原因死亡的时间。次要结局指标为胸膜自发固定，定义为在未使用胸膜硬化剂的情况下，经过连续3天的评估，胸腔引流均达到最小引流量（<50 mL/d），并且通过X线、胸部CT或者超声检查证实PE减少后，拔除胸腔引流管。

### 1.4 统计学方法

应用SPSS version 26.0和Rversion 4.2.3进行统计分析，正态分布的计量资料采用均数±标准差描述，非正态分布的计量资料采用中位数（四分位数间距）描述。频率和百分比用于描述分类变量，组间比较采用卡方检验。应用Kaplan-Meier法进行生存分析，采用Log-rank检验进行组间比较。应用Nelson-Aalen累积风险曲线分析患者胸膜自发固定的发生率。使用Cox比例风险模型进行OS和胸膜自发固定的单变量和多变量回归分析，单因素分析中*P*<0.2的变量被纳入多变量模型中，计算调整后的风险比（hazard ratio, HR）和95%置信区间（confidence interval, CI）。采用受试者工作特征（receiver operating characteristic, ROC）曲线评估模型的预测效能。P值为双侧检验，当*P*<0.05表示差异有统计学意义。

## 2 结果

### 2.1 患者的临床特征

根据纳入与排除标准本研究总计纳入261例晚期NSCLC伴MPE患者，中位年龄为62.3（52.7, 70.0）岁，65岁及以下占58.2%（152/261）；男性占53.6%（140/261）；有吸烟史占34.1%（89/261）；腺癌占95.8%（250/261）；ECOG PS评分为0-1分占80.5%（210/261）。随后按照6:4比例随机抽取157例作为训练集，余下104例作为验证集，两组患者在年龄、性别、吸烟史、病理类型和ECOG PS评分等方面未显示统计学差异（*P*<0.05）（表 1）。

**表 1:** NSCLC伴恶性胸腔患者的基线特征

Clinical characteristics	Total (*n*=261)	Development set (*n*=157)	Validation set (*n*=104)	P
Age (yr)				0.452
<65	152 (58.2%)	88 (56.1%)	64 (61.5%)	
≥65	109 (41.8%)	69 (43.9%)	40 (38.5%)	
Gender				0.405
Female	121 (46.4%)	69 (43.9%)	52 (50.0%)	
Male	140 (53.6%)	88 (56.1%)	52 (50.0%)	
Smoking status				0.999
Current/ex-smoker	89 (34.1%)	54 (34.4%)	35 (33.7%)	
Never smoker	172 (65.9%)	103 (65.6%)	69 (66.3%)	
Pathological type				0.941
Adenocarcinoma	250 (95.8%)	151 (96.2%)	99 (95.2%)	
Squamous cell carcinoma	11 (4.2%)	6 (3.8%)	5 (4.8%)	
BMI (kg/m²)				0.527
<18.5	24 (9.2%)	14 (8.9%)	10 (9.6%)	
18.5-23.9	143 (54.8%)	82 (52.2%)	61 (58.7%)	
24.0-27.9	81 (31.0%)	54 (34.4%)	27 (25.9%)	
≥28.0	13 (5.0%)	7 (4.5%)	6 (5.8%)	
ECOG performance status				0.183
0-1	210 (80.5%)	131 (83.4%)	79 (76.0%)	
2-4	51 (19.5%)	26 (16.6%)	25 (24.0%)	
T stage				0.638
T1	31 (11.9%)	21 (13.4%)	10 (9.6%)	
T2-T3	108 (41.4%)	63 (40.1%)	45 (43.3%)	
T4	122 (46.7%)	73 (46.5%)	49 (47.1%)	
N stage				0.999
N0-N1	39 (14.9%)	23 (14.6%)	16 (15.4%)	
N2-N3	222 (85.1%)	134 (85.4%)	88 (84.6%)	
Distant organ metastasis number				0.907
<2	198 (75.9%)	120 (76.4%)	78 (75.0%)	
≥2	63 (24.1%)	37 (23.6%)	26 (25.0%)	
Actionable genetic alterations				0.937
EGFR/ALK/ROS1 wt	76 (29.1%)	48 (30.6%)	28 (26.9%)	
ALK/ROS1 fusion	25 (9.6%)	15 (9.5%)	10 (9.6%)	
EGFR mutation	160 (61.3%)	94 (59.9%)	66 (63.5%)	

NSCLC: non-small cell lung cancer; MPE: malignant pleural effusion; BMI: body mass index; ECOG: Eastern Cooperative Oncology Group; EGFR: epidermal growth factor receptor; ALK: anaplastic lymphoma kinase; ROS1: ROS proto-oncogene 1, receptor tyrosine kinase; wt: wild type.

### 2.2 全身炎症系数的定义及界值

本文分析中使用全身炎症系数包括：ALI: [白蛋白（g/L）/中性粒细胞与淋巴细胞比率]×身体质量指数（body mass index, BMI）（kg/m²）^[13]^、PNI: [外周血淋巴细胞总数×0.005＋血清白蛋白（g/L）]^[14]^、SII: [（血小板计数×中性粒细胞计数）/淋巴细胞计数]/10^[16]^、HALP: [血红蛋白（g/L）×白蛋白（g/L）×淋巴细胞计数/血小板计数]^[15]^。通过计算最大约登指数，在训练集中对全身炎症系数进行二分类，具体如下：ALI: 274.1；PNI: 44.1；SII: 106.8；HALP: 31.8。

### 2.3 全身炎症系数在NSCLC伴MPE患者的预后中的比较

进一步探索上述全身炎症系数在NSCLC伴MPE患者预后中的差异。通过使用最大约登指数将连续变量PNI二分类，其中PNI<44.1患者70例，PNI≥44.1患者87例。OS的Kaplan-Meier生存分析结果显示，PNI<44.1组中位OS为24.3个月（95%CI: 20.4-40.8），PNI≥44.1的患者中位OS为36.5个月（95%CI: 30.8-54.6），差异具有统计学意义（*P*=0.02，图1A）。在EGFR突变的亚组中，与PNI<44.1的患者相比，PNI≥44.1的患者中位OS显著延长（36.5 vs 23.1个月，*P*=0.014）。此外，不同ALI、SII、HALP组间中位OS差异无统计学意义（*P*>0.05）（图1B-1D）。

**图 1:**
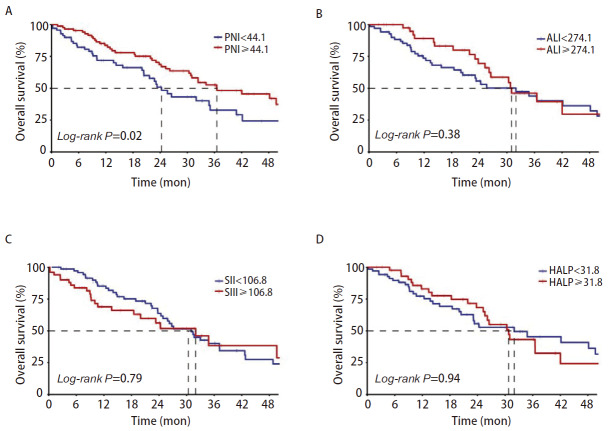
训练集中晚期NSCLC伴MPE患者OS的Kaplan-Meier曲线。A：比较PNI<44.1和PNI≥44.1组患者的Kaplan-Meier生存曲线；B：比较ALI<274.1和ALI≥274.1组患者的Kaplan-Meier生存曲线；C：比较SII<106.8和SII≥106.8组患者的Kaplan-Meier生存曲线；D：比较HAL*P*<31.8和HALP≥31.8组患者的Kaplan-Meier生存曲线。

### 2.4 NSCLC伴MPE患者预后的Cox比例风险模型分析

通过OS的单因素Cox比例风险模型（表2）分析得出，年龄≥65岁、原发肿瘤≥5 cm可能与NSCLC伴MPE患者的不良预后有关，而PNI≥44.1、ALK/ROS1融合可能与NSCLC伴MPE患者的预后良好有关（*P*<0.20）；进一步多因素Cox回归分析发现，PNI是影响NSCLC伴MPE患者预后的独立影响因素（HR=0.449, 95%CI: 0.220-0.916, *P*=0.028），年龄≥ 65岁（HR=1.644, 95%CI: 1.009-2.680, *P*=0.046）和原发肿瘤 ≥5 cm（HR=2.179, 95%CI: 1.246-3.808, *P*=0.006）与NSCLC伴MPE患者不良预后有关。

**表 2:** 训练集中晚期NSCLC伴MPE患者OS的单因素与多因素Cox回归分析

Characteristics	n (%)	Univariate analysis			Multivariate analysis	
		HR (95%CI)	P		HR (95%CI)	P
Age (yr)						
<65	88 (56.1)	1.000 (Reference)			1.000 (Reference)	
≥65	69 (43.9)	2.105 (1.336-3.316)	0.001		1.644 (1.009-2.680)	0.046
Smoking status						
Never smoker	103 (65.6)	1.000 (Reference)				
Current/ex-smoker	54 (34.4)	0.886 (0.539-1.461)	0.634			
ECOG PS						
0-1	131 (83.4)	1.000 (Reference)				
2-4	26 (16.6)	1.296 (0.734-2.286)	0.372			
BMI (kg/m²)						
<18.5	14 (8.9)	1.000 (Reference)				
18.5-23.9	82 (52.2)	0.486 (0.103-2.298)	0.362			
24.0-27.9	54 (34.4)	0.913 (0.425-1.961)	0.816			
≥28.0	7 (4.5)	0.946 (0.429-2.086)	0.890			
Primary tumor size (cm)						
<5	102 (65.0)	1.000 (Reference)			1.000 (Reference)	
≥5	27 (17.2)	2.336 (1.341-4.068)	0.003		2.179 (1.246-3.808)	0.006
Unmeasurable	28 (17.8)	2.144 (1.220-3.767)	0.008		1.946 (1.096-3.455)	0.023
Distant organ metastasis number						
<2	120 (76.4)	1.000 (Reference)				
≥2	37 (23.6)	1.138 (0.669-1.936)	0.632			
Actionable genetic alterations						
EGFR/ALK/ROS1 wt	48 (30.6)	1.000 (Reference)			1.000 (Reference)	
ALK/ROS1 fusion	15 (9.6)	0.499 (0.199-1.251)	0.138		0.565 (0.210-1.519)	0.258
EGFR mutation	94 (59.9)	1.152 (0.685-1.936)	0.594		1.190 (0.700-2.022)	0.521
Pleural fluid volume						
Little	26 (16.6)	1.000 (Reference)				
Large	131 (83.4)	0.747 (0.410-1.359)	0.339			
Trapped lung						
No	69 (43.9)	1.000 (Reference)				
Yes	88 (56.1)	0.882 (0.512-1.505)	0.644			
Septated pleura effusion						
No	141 (89.8)	1.000 (Reference)				
Yes	16 (10.2)	0.912 (0.388-2.144)	0.832			
PNI						
<44.1	70 (44.6)	1.000 (Reference)			1.000 (Reference)	
≥44.1	87 (55.4)	0.433 (0.215-0.869)	0.019		0.449 (0.220-0.916)	0.028

HR: hazard ratio; CI: confidence interval.

### 2.5 PNI在预测MPE患者胸膜自发固定中的应用

上述预后的多因素Cox回归分析发现PNI是MPE患者不良预后的独立影响因素，进一步探究PNI在预测接受胸腔引流管置入的MPE患者胸膜自发固定中的作用。与PNI<44.1的患者相比，PNI≥44.1的患者60天胸膜自发固定发生率高（90.4% vs 77.3%），差异具有统计学意义（*P*=0.009，图2）。

**图 2:**
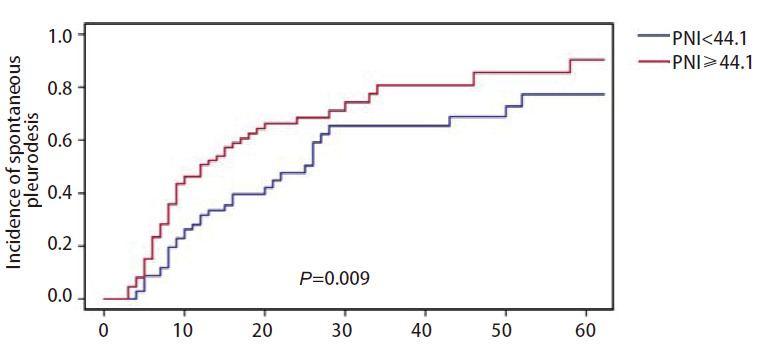
比较训练集中PNI<44.1和PNI≥44.1的晚期NSCLC伴MPE患者胸膜自发固定率

### 2.6 NSCLC伴MPE患者胸膜自发固定的Cox比例风险模型分析

关于胸膜自发固定的单因素Cox比例风险模型（表3）分析得出，PNI≥44.1、鳞癌与患者胸膜自发固定的发生可能有关联，而大量PE、肺不张可能与患者胸膜自发固定失败有关（*P*<0.20）；进一步多因素Cox回归分析发现，PNI≥44.1是NSCLC伴MPE患者发生胸膜固定的独立影响因素（HR=1.641, 95%CI: 1.077-2.502, *P*=0.021），鳞癌（HR=2.438, 95%CI: 1.040-5.712, *P*=0.040）对胸膜自发固定成功有影响，而大量PE（HR=0.415, 95%CI: 0.251-0.683, *P*<0.001）与胸膜自发固定失败有关联。

**表 3:** 训练集晚期NSCLC伴MPE患者胸膜自发固定的单因素与多因素Cox回归分析

Characteristics	n (%)	Univariate analysis			Multivariate analysis	
		HR (95%CI)	P		HR (95%CI)	P
Age (yr)						
<65	88 (56.1)	1.000 (Reference)				
≥65	69 (43.9)	0.990 (0.658-1.489)	0.962			
ECOG PS						
0-1	131 (83.4)	1.000 (Reference)				
2-4	26 (16.6)	0.991 (0.570-1.722)	0.974			
BMI (kg/m²)						
<18.5	14 (8.9)	1.000 (Reference)				
18.5-23.9	82 (52.2)	0.832 (0.283-2.449)	0.739			
24.0-27.9	54 (34.4)	0.924 (0.468-1.825)	0.821			
≥28.0	7 (4.5)	0.601 (0.291-1.240)	0.168			
Primary tumor size (cm)						
<5	102 (65.0)	1.000 (Reference)				
≥5	27 (17.2)	0.983 (0.573-1.686)	0.951			
Unmeasurable	28 (17.8)	0.573 (0.319-1.027)	0.062			
Type of pathology						
Adenocarcinoma	151 (96.2)	1.000 (Reference)			1.000 (Reference)	
Squamous cell carcinoma	6 (3.8)	2.221 (0.965-5.112)	0.061		2.438 (1.040-5.712)	0.040
Actionable genetic alterations						
EGFR/ALK/ROS1 wt	48 (30.6)	1.000 (Reference)				
ALK/ROS1 fusion	15 (9.6)	0.815 (0.388-1.713)	0.589			
EGFR mutation	94 (59.9)	0.747 (0.477-1.169)	0.202			
Pleural fluid volume						
Little	26 (16.6)	1.000 (Reference)			1.000 (Reference)	
Large	131 (83.4)	0.417 (0.255-0.682)	<0.001		0.415 (0.251-0.683)	<0.001
Trapped lung						
No	69 (43.9)	1.000 (Reference)			1.000 (Reference)	
Yes	88 (56.1)	0.738 (0.491-1.108)	0.142		0.889 (0.587-1.348)	0.579
Septated pleura effusion						
No	141 (89.8)	1.000 (Reference)				
Yes	16 (10.2)	0.623 (0.272-1.460)	0.281			
Thoracoscopic treatment						
No	89 (93.0)	1.000 (Reference)				
Yes	11 (7.0)	1.461 (0.795-2.684)	0.222			
PNI						
<44.1	70 (44.6)	1.000 (Reference)			1.000 (Reference)	
≥44.1	87 (55.4)	1.733 (1.140-2.633)	0.010		1.641 (1.077-2.502)	0.021

### 2.7 PNI预测模型的验证

对建立PNI相关的预后和胸膜自发固定模型进行内部数据集的验证，并使用ROC曲线评估模型的预测性能。PNI-预后模型能够较好地预测晚期肺癌伴MPE患者的预后，训练集和验证集ROC曲线下面积（area under the curve, AUC）分别为0.694（95%CI: 0.620-0.776）和0.657（95%CI: 0.551-0.762）（图3A）。PNI-胸膜自发固定模型预测训练集和验证集的AUC分别为0.673（95%CI: 0.590-0.737）和0.691（95%CI: 0.573-0.808），表明该模型具有良好的预测能力（图3B）。

**图 3:**
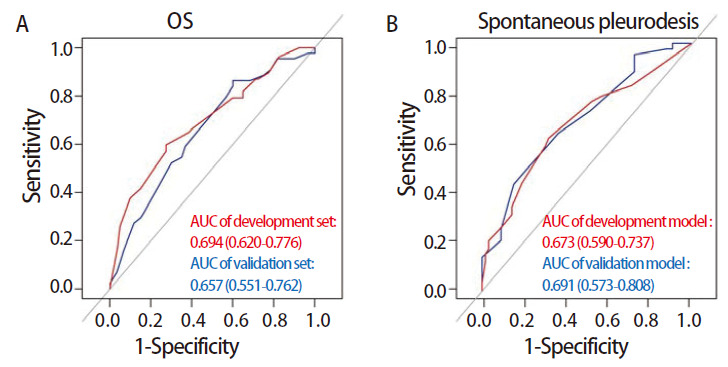
基于多因素Cox回归结果绘制的ROC曲线。A：基于OS多因素Cox分析结果的ROC曲线；B：基于胸膜自发固定多因素Cox分析结果的ROC曲线。

## 3 讨论

本研究中回顾了261例晚期NSCLC伴症状性MPE患者，分析PNI与患者OS和胸膜自发固定的相关性。PNI反映了患者营养状态、肿瘤微环境及免疫状态，具有预测MPE患者胸膜自发固定成功和长期生存的能力。

本研究中PNI能够更好地预测MPE整体人群患者的预后，同时在接受EGFR-酪氨酸激酶抑制剂（tyrosine kinase inhibitors, TKIs）治疗的亚组中也具有良好的预测能力。由于ALK/ROS1融合及驱动基因阴性的患者较少，尚未探究PNI在该亚组中的作用。白蛋白能够反映机体的营养储备，具有调节免疫功能和平衡体液的作用。既往的研究^[7,17]^已证实长期的营养不良、恶病质与肿瘤患者较差的预后有关。较低的血清白蛋白水平与肿瘤进展相关，从而导致MPE患者生存时间缩短^[18,19]^。而营养补充品可抑制髓源性抑制细胞在患者体内的募集，降低白介素-6表达，进而减缓头颈癌的增殖^[20]^。肿瘤微环境中的淋巴细胞是适应性免疫的重要组成部分，通过分泌抑制性细胞因子和诱导细胞毒性细胞死亡来抑制肿瘤生长和转移^[21]^。当淋巴细胞数量减少时，免疫系统抑制肿瘤细胞生长和转移的能力下降，进而导致肿瘤的进展^[22]^。据报道^[23⇓-25]^，淋巴细胞的减少、中性粒细胞增多与MPE患者较差的预后和胸膜自发固定失败有关。PNI是结合血清白蛋白及外周血淋巴细胞数计算所得，是肿瘤的重要预后因素^[14,26]^。既往研究^[26]^报道，相较于炎症或营养预测模型，联合模型将能更准确地预测结直肠癌患者OS（AUC: 0.796 vs 0.793 vs 0.820）。此外，在探究晚期食管鳞癌患者不良预后影响因素时，研究者将近1个月内是否使用抗生素纳入多因素Cox分析，发现基线PNI仍然是患者不良预后的独立影响因素^[27]^。PNI的动态变化能够较好预测晚期肺癌患者的预后和骨转移灶的反应率^[28,29]^。PNI在预测转移性胃癌预后效能中优于中性粒细胞与淋巴细胞计数比值、血小板与淋巴细胞计数比值和SII等血液学指标，能够更好地预测胃癌患者的OS和无病生存期^[30]^。

本研究评估了PNI在MPE患者胸膜自发固定中的作用。研究^[31,32]^表明血清或PE中白蛋白水平能够预测胸膜自发固定的发生。全身炎症反应的激活（C反应蛋白升高）是胸膜固定成功的预测因素^[33,34]^，淋巴细胞的减少、中性粒细胞增多与MPE患者胸膜自发固定失败有关^[33,35,36]^。因此，较高PNI的患者可能因其具有较高白蛋白和淋巴细胞，更容易发生胸膜自发固定。此外，相关研究^[37,38]^表明，与胸膜固定失败的患者相比，胸膜固定成功的患者生存时间延长，这可能后者具有更好的MPE控制率和炎症反应有关^[39⇓-41]^。对于胸膜固定失败的患者，大量MPE及其包含的细胞因子可能会促进肿瘤细胞的增殖、侵袭，进而影响抗肿瘤治疗的疗效^[39,40]^。胸膜固定成功的患者中，C反应蛋白升高表明炎症反应被激活，有助于免疫细胞更好地发挥抗肿瘤功能^[41]^。因此，PNI作为反映全身营养和免疫状态的指标，能够更全面、准确地预测MPE患者的预后和胸膜自发固定的发生。

本研究的局限性主要有以下3点：（1）本研究对单中心晚期NSCLC伴MPE患者数据进行分析，回顾性研究依赖于分析数据的质量和缺失程度，这可能导致数据异质性较大，进而影响OS的结果；（2）本研究中将连续变量PNI等进行二分类，虽然更容易解释并临床应用，但也削弱了变量的预测能力，另外PNI是动态变化的指标，连续监测或具有更精确的预测效能；（3）常规收集临床检验数据可能会受到患者实际状态的影响，例如患者处于急性炎症状态以及在确诊前1个月内曾接受抗生素治疗，当前尚不清楚这是否会为患者带来更大的疾病负担，因此，本研究在多变量分析中纳入原发肿瘤相关因素等作为分类变量试图以减轻此类影响。尽管如此，本研究仍具有较大的意义，未来的工作应旨在排除此类偏倚来源，基于前瞻性、多中心和大样本数据进行进一步的研究。本研究通过对晚期肺癌合并MPE患者的临床数据分析发现，较高的PNI是MPE患者长期生存和胸膜自发固定的独立影响因素，可为MPE患者生存时间的评估和治疗方案的选择提供理论参考。
